# Ultra-processed food consumption in Scotland: a nationally representative analysis of sociodemographic patterns and dietary contributions

**DOI:** 10.1017/S1368980026102559

**Published:** 2026-04-30

**Authors:** Josephine Mary Curtis, Nick Townsend

**Affiliations:** University of Bristol, UK

**Keywords:** Ultra-processed foods, NOVA classification, Sociodemographic disparities, Public health policy

## Abstract

**Objective:**

To quantify ultra-processed food (UPF) intake in Scotland, identify key contributing food groups and examine sociodemographic associations using nationally representative data.

**Design:**

Cross-sectional analysis of 2021 Scottish Health Survey data using 2-d dietary recalls via Intake24 classified by NOVA. UPF intake was calculated as percentage of total energy intake (%TEI) and grams per day (g/d). Multivariable linear regression assessed associations with sex, age, ethnicity, income, socio-economic classification, highest educational qualification, urban–rural location, region and Scottish Index of Multiple Deprivation (SIMD) quintiles.

**Setting:**

Nationally representative sample of Scottish households.

**Participants:**

Individuals aged 16 years or over with complete dietary and sociodemographic data (*n* 2645).

**Results:**

Mean energy intake was 1637·8 kcal/d (95 % CI 1615·8, 1659·8). Mean UPF consumption was 666·9 g/d (95 % CI 647·9, 685·9), amounting to 919·9 kcal/d (95 % CI 901·1, 938·6), representing 55·4 % of TEI (95 % CI 54·7, 56·2) and 28·2 % of total food weight. The main contributors to UPF intake were cereal products (244·8 kcal/d, 27·0 % of UPF kcal), confectionery (170·3 kcal/d, 17·9 %) and meats (153·6 kcal/d, 16·2 %). Sandwiches (99·9 % UPF), salty snacks (94·1 %) and dietary supplements (90·5 %) showed highest UPF proportions by food groups. Adjusted analyses revealed greater UPF consumption (%TEI) among males (*β* = −3·3, *P* < 0·001), younger adults (*β* = −2·8 per decade, *P* < 0·001), White participants (*β* = +12·9 *v*. non-White, *P* < 0·001) and lower SIMD quintile (*β* = −1·8 per quintile, *P* < 0·001). Similar patterns emerged for absolute intake (g/d).

**Conclusions:**

UPF dominates Scotland’s diet, with inequitable distribution across sociodemographic groups. Policy actions – such as adopting NOVA in dietary guidelines and restricting UPF marketing – are urgently needed to address this public health crisis.

Over the past 40 years, the way in which the world consumes food has radically changed. We have witnessed a ‘nutrition transition’ away from obtaining sustenance from minimally processed food (MPF) to ‘ultra-processed food’ (UPF)^(1)^. UPF is industrially formulated from whole foods, typically containing cosmetic additives such as flavours, colours and emulsifiers^(2)^. Often high in added salt, sugar and fat, UPF constitutes a substantial proportion of the modern Western diet, including breakfast cereals, sugary drinks, ready meals and mass-produced confectionery and savoury snacks^(3)^.

UPF tends to displace MPF in diets since it is often hyper-palatable, durable and inexpensive, meaning individuals may choose UPF as it can be tastier, cheaper, has a longer shelf life and is more convenient^(4)^. However, food choices are also highly influenced by wider socio-economic and cultural factors, something that Dahlgren and Whitehead’s ‘social determinants of health’ model demonstrated in 1991^(5)^.

Rising incomes, urbanisation, the increase in female formal employment and an accompanying decline in cooking skills have propelled the consumption of UPF, first within high-income countries (HIC), but more recently within low-to-middle-income countries^(1,6)^. Reviews have found that the rate of UPF consumption as a percentage of daily energy is growing at a faster rate in low-to-middle-income countries than HIC^(6,7)^.

But attention must also be paid to the role of ‘Big Food’: transnational UPF corporations who seek to maximise profits through aggressive mass-marketing campaigns^(7)^. Studies have found that such campaigns are often directed at younger and more deprived individuals^(8,9)^.

Accompanying this ‘nutrition transition’ has been a doubling in global rates of obesity since 1990, with over 2·5 billion adults now classified as overweight or obese^(10)^. Furthermore, rates of diet-related non-communicable diseases, such as heart disease, stroke, diabetes and cancer, have rapidly accelerated. Non-communicable diseases now account for almost three-quarters of global deaths^(11)^.

A systematic review has found an overall positive correlation between a higher consumption of UPF and a higher incidence of obesity^(12)^. UPF consumption has also been associated with higher rates of cancer^(13)^, dementia^(14)^, diabetes^(15)^, CVD^(16)^ and among other diseases. As a potential explanation, correlations have been found between high UPF intake and a diet high in sugar, saturated fat and carbohydrates but low in protein, fibre and vitamins^(2,17)^. Aside from its impact on human health, the production and consumption of UPF accounts for up to one-third of total diet-related greenhouse gas emissions, land use and food waste and between 36 and 45 % of total diet-related biodiversity loss^(18)^.

Certain regions have taken notice of these findings, principally throughout South America, in which avoidance of UPF is various national governments’ ‘golden rule’ of public health advice^(3,19)^. However, some governments – notably the UK – have chosen not to incorporate limits on UPF into their official nutrition guidance^(20)^.

Providing further impetus for such policy changes are studies which aim to uncover the extent of UPF consumption across different contexts. A systematic review collated data from nationally representative studies across thirty-two countries which used food consumption tools such as 24-h dietary recalls or FFQ to monitor dietary intake, finding that average UPF consumption as a percentage of total energy intake (%TEI) ranges from a low of 14–16 % in Italy, Romania and Colombia to a high of 61 % in the Netherlands^(19)^. The studies reviewed also assessed UPF consumption according to sociodemographic measures^(21)^. Across all studies, a younger age, being unmarried and living in an urban area were associated with a higher UPF consumption.

Within the UK, studies have shown that UPF comprises between 56·8 and 63 % of average TEI^(2,22)^. Significant associations have been found between UPF intake and a younger age, male gender, low socio-economic status and more deprived indices of multiple deprivation quintile^(22,23)^. This indicates that, in addition to high overall consumption levels, UPF intake in the UK varies significantly across different sociodemographic groups. These disparities highlight the need for targeted policy interventions that address the specific barriers and challenges faced by vulnerable populations.

However, studies on the Scottish population are lacking. This is despite Scotland having the highest rates of obesity and CVD-related deaths of any UK nation, accompanied by profound, worsening health inequalities^(24–26)^. By 2021, almost 70 % of Scottish adults were overweight or obese, with obesity prevalence at 38 % in the most deprived areas compared with 22 % in the least deprived, meaning that since 2012, the deprivation gap doubled from 8 % to 16 %^(24)^.

Poor diet is considered a driving factor behind this, and negative stereotypes of the Scottish diet – that it consists of unhealthy deep-fried foods – is widely propagated in the media^(26)^.

Previous quantitative studies have analysed food purchase data in Scotland, finding a higher energy density in foods purchased by the most deprived households^(27,28)^. Another analysed food consumption questionnaires within the Scottish Health Surveys 1995, 1998 and 2003, finding that Glasgow had an ‘unfavourable dietary profile’ compared to other areas of the country^(29)^.

In response to Scotland’s poor health profile, the Scottish Government has introduced numerous dietary targets since 1996, none of which have been met^(30)^. This is perhaps unsurprising, since the government’s attempts to promote ‘healthy’ eating can be contradictory (e.g. the Government-owned Parent Club website provides children’s recipes involving products that the Government’s ‘Eatwell Guide’ and dietary goals advise against, such as processed meats)^(31,32)^.

In July 2022, the government consulted to limit the sales of foods high in fat, salt and sugar (HFSS). These were labelled ‘discretionary products’ – such as ‘savoury snacks’, ‘sweet biscuits’ and ‘dairy desserts’ – and ‘additional categories’ – such as ‘fromage frais’, ‘breakfast cereals’ and ‘roast potatoes, chips, and similar potato products’^(33)^.

To support these measures, the government commissioned an analysis from Food Standards Scotland (FSS) of the food diaries of participants of the Scottish Health Survey 2021. FSS found that discretionary food consumption equated to 28 % of TEI and was highest in men and in areas of high deprivation^(33)^. Yet, there was a positive correlation between an older age and intake of this food category^(33)^. Discovering that it is the over-75 age category who consume the most ‘discretionary foods’ exposes either that sociodemographic-related UPF consumption in Scotland is radically dissimilar to other countries or that the Scottish Government’s classification of unhealthy foods is distorted, both with significant policy implications.

Ultimately, no study has examined UPF consumption using a nationally representative sample of the Scottish population. Therefore, this study’s research questions were as follows:

1. What are the levels of UPF consumption across Scotland?
2. Which food categories contribute the most to overall UPF consumption?
3. Are sociodemographic factors associated with the consumption of UPF?

## Methods

We conducted an analysis using cross-sectional data from participants aged 16 years and over in the Scottish Health Survey 2021 (SHeS21). Data were downloaded from the UK Data Service^(34)^. Direct consent was obtained for all participants for the SHeS21. Further ethical approval for this secondary analysis of anonymised data was not required.

### Study design and population

SHeS21 is an annual cross-sectional survey conducted by the Scottish Centre for Social Research (ScotCen) and funded by the Scottish Government^(35)^. It assesses participants’ health through various measures including smoking prevalence, alcohol consumption, physical activity, and diet.

Since it was designed to be representative of the total population, the survey used a partially clustered, stratified, multistage sampling technique. Addresses were selected from the Postcode Address File and provided with postal communication asking them to opt in to the study.

SHeS21 data collection ran from April to December 2021. Participants were interviewed via telephone, and they filled out either an online or paper self-completion questionnaire. Participants aged 16 years and above were also asked to complete a 2-d food recall diary using Intake24 either via an app or through a telephone call with the University of Cambridge study team. The days selected for recall were randomised over the course of 1 week. Further information on sampling can be found elsewhere^(36)^.

### Dietary data collection and processing

Intake24 is an ‘open-source, self-completed, computerised dietary recall system’^(37)^. It contains a database of over 2800 foods, is receptive to spelling errors, uses image-based portion size estimation and takes on average under 20 min to complete. It also asks further prompts after a user enters a food item such as ‘Did you have milk in your coffee?’, enhancing its accuracy.

Daily and seasonal variation in food intake is addressed by the continuous fieldwork design and the requirement of two randomised days of recall.

The food lists and nutrient databank on Intake24 are aligned with the UK National Diet and Nutrition Survey (NDNS), allowing for comparisons with other studies using this database^(37)^.

### Inclusion criteria

Individuals were included in the present study if they took part in SHeS21, were aged 16 years or over, completed 2 d of dietary recall via Intake24 and answered all questions relating to the sociodemographic variables of interest.

Eligibility for dietary recall analysis was defined by the Scottish Health Survey protocol, including the identification and review of incomplete recalls and extreme outliers. Recalls containing ≤ 10 items, durations ≤ 2 min or < 400 kcal were reviewed on a case-by-case basis and excluded if deemed incomplete. Extreme outliers in portion sizes, energy and nutrient intakes (> 3 × IQR) were identified using boxplots and reviewed individually, and implausible values were adjusted where appropriate^(38)^.

No participants were excluded on the basis of pregnancy, lactation or medical conditions affecting diet. Expressing UPF intake as a percentage of TEI may have partially mitigated the variability introduced by these factors.

### NOVA classification

Classification of food and beverage items was conducted using the NOVA classification system. NOVA, first proposed by researchers at the University of São Paolo in 2009 and refined in 2016, groups foods into four categories based on the extent and purpose of processing: (1) unprocessed foods or MPF, (2) processed culinary ingredients, (3) processed foods and (4) UPF. Table 1 describes these four categories and offers examples of each. Further details on the NOVA classification can be found elsewhere^(3)^.

**Table 1.** The NOVA classification system of food processing, alongside examples

Group 1 – unprocessed/minimally processed foods	Group 2 – processed culinary ingredients
Includes edible parts of plants, animals, fungi and algae, or whole foods processed (e.g. dried, frozen, roasted, pasteurised) to extend shelf life without adding ingredients. Examples: fruits, vegetables, meat, pasta, plain yogurt.	Derived from Group 1 foods through processes like refining, milling or pressing, used in cooking. Rarely consumed alone. Examples: salt, sugar, oils, honey, butter, starches.
Group 3 – Processed foods	Group 4 – Ultra-processed foods
Combinations of Group 1 and 2 foods, often with added salt, sugar or oil to enhance shelf life or taste. May include preservatives. Examples: tinned vegetables, salted nuts, cured meats, cheese, fresh bread, fruits in syrup.	Industrial formulations with minimal Group 1 foods, containing additives like flavour enhancers or anti-caking agents. Processes include extrusion or pre-frying to create ready-to-eat, profitable products. Examples: carbonated drinks, breakfast cereals, confectionery, crisps, ready meals.

It is important to note that NOVA is a framework for classifying foods by processing level rather than by healthfulness. Not all UPF is inherently unhealthy – for example, infant formula and supplements can contribute positively to diets – and many less healthy foods, such as cured meats and sweet baked goods, fall outside Group 4. This distinction is essential for interpreting studies of UPF consumption, as they measure exposure to processing rather than direct diet quality.

For the purposes of this analysis, Groups 1–3 (MPF, processed culinary ingredients and processed foods) were combined and treated as ‘non-UPF’. This binary classification (UPF *v*. non-UPF) was chosen to focus specifically on UPF consumption, consistent with previous research using national dietary datasets^(39)^.

Initially, JC manually coded each food or drink item in the dataset containing all respondents’ food diaries as either 1 for UPF (Group 4) or 0 for non-UPF (Group 1, 2 and 3). NT verified the NOVA classifications, and initial agreement was high (> 95 %). In cases of disagreement or uncertainty regarding certain items, additional resources were consulted until full consensus was achieved. First, we consulted supermarket websites to analyse ingredients lists of popular branded items. Open Food Facts was also useful for this purpose^(40)^. In addition, we drew upon previous research which analysed data from the NDNS to act as a coding framework^(2,4)^.

### Variables of interest

#### Outcome variables

The primary outcomes of interest were the percentage of TEI derived from UPF (%TEI/d) and the total weight of UPF consumed (g/d). These metrics were determined by analysing the relative energy contribution and the total weight of foods and beverages classified as UPF in the Intake24 dietary dataset, as reported by each participant, based on the mean of two 24-h dietary recalls.

#### Sociodemographic variables

Sociodemographic characteristics of interest included sex, age, ethnicity, income, socio-economic classification, highest educational qualification, Scottish Index of Multiple Deprivation (SIMD) quintile, urban–rural location and region.

We used SHeS21 sociodemographic variables with many directly lifted. Sex (SHeS21 = Sex) was categorised as male or female. Age (SHeS21 = ag16g10) was presented as seven categories in 10-year bands (16–24 – 75+). We recoded ethnicity (SHeS21 = Ethnic05) from five (White Scottish, White British, White Other, Asian and Other Minority Ethnic) to two (White and Non-White) categories to enhance their statistical power. Income (SHeS21 = eqv5_15) was measured along quintiles using the Organisation for Economic Co-operation and Development (OECD) equivalisation scale to account for differences in household size. Socio-economic classification (SHeS21 = hpnssec3) was measured using tertiles of NS-SEC, the ‘National Statistics Socio-economic Classification’, a measure of social class based upon employment relations and conditions of occupation (higher managerial, administrative and professional occupations; intermediate occupations; routine and manual occupations). However, full-time students and the long-term unemployed are not included in this classification and therefore present as ‘not applicable’^(41)^.

Highest educational qualification (SHeS21 = hedqul08) was measured as: degree or higher; Higher National Certificate/Diploma (HNC/D) or equivalent; Higher grade or equivalent; Standard grade or equivalent; other school level; or no qualifications. SIMD (SHeS21 = simd20_sga), urban–rural location (SHeS21 = urbrur2a_20) and region (SHeS21 = HBCode) were determined from a participant’s postcode. Full explanations of how SIMD and urban–rural location are calculated can be found elsewhere^(42,43)^. For our analysis, we recoded SHeS21’s ‘Health Board Code’ variable (which comprises fourteen areas) into three regions: North, East and West, informed by other studies^(44)^.

All variables apart from sex were derived variables, meaning that they are either recodes of existing variables or they draw upon several variables to make new variables^(35)^. ScotCen advise using derived variables in analysis when there are multiple measures of the same variable in the dataset.

#### Food group variables

Before calculating UPF consumption according to food groups, the food groups provided by SHeS21 first had to be recoded. SHeS21’s 62 food groups were aggregated into 18. A similar study examining UPF consumption from a Swiss national dietary survey was used as a reference point^(21)^. Categories were combined on the basis of nutritional and culinary similarity (e.g. all milks under ‘Dairy products’; all meats and fish under ‘Meat, fish, and eggs’), while existing composite groups such as ‘Sandwiches’ were retained. Subcategories of SHeS food group 50 – ‘Miscellaneous’ – were assigned to soups, sauces, beverages or supplements as appropriate, and toddler foods (SHeS food group 52) were individually coded into new food groups. The full mapping is shown in online Supplementary Table 1.

### Statistical analysis

Study weights were provided by SHeS21 to account for the probability of selection and non-response, producing estimates representative of the Scottish population. The Intake24 weighting variable was applied in all analyses.

Descriptive statistics are presented as means and standard deviations of UPF intake (%TEI/d and g/d) overall and by sociodemographic group. Associations between UPF consumption and sociodemographic characteristics were examined using multivariable linear regression models. Assumptions of linear regression were assessed, including linearity, homoscedasticity, absence of multicollinearity (using variance inflation factors) and normality of residuals using histograms and Q–Q plots. Although linear regression assumes normally distributed residuals, under the central limit theorem it is considered robust to deviations from normality in large samples, consistent with the sample size in the present study.

Variables for Sex, Ethnicity and Urban/Rural location were binary. Reference categories for these have been provided in the table. Region was aggregated into a nominal variable with three regions: (1) Northern, (2) Eastern and (3) Western; with Western used as the reference category. The remaining variables, such as Age, Income Socioeconomic classification, Highest educational qualification, and SIMD quintiles, were included as ordinal variables.

All data analysis was conducted using IBM SPSS Statistics, version 30.0.0.0.

### Missing data

Four hundred and five (405) incomplete dietary recalls (1 d provided instead of 2) were returned. This resulted in 3042 participants remaining.

Sociodemographic variables with complete data were sex, age, urban–rural location, SIMD quintile and region. Variables with missing data (non-weighted) were ethnicity (0·1 %), income (8·5 %), socio-economic classification (1·1 %) and highest educational qualification (0·2 %). A ‘not applicable’ outcome in the derived variables of income and socio-economic classification was a result of internal routing errors (incomplete data elsewhere)^(35)^.

A complete-case analysis was performed (non-weighted *n* 2759).

## Results

### Characteristics of participants

After applying the SHeS21 Intake24 scaling weight, the total number of participants was 2645. As Table 2 shows, the most represented participants were women (52·6 %), ages 45–54 years (17·5 %), living in the Western region (49·9 %), in urban (83·1 %) and the least deprived areas (SIMD quintile 5 – 21·8 %), of White ethnicity (94·5 %) and a managerial or professional socio-economic classification (55·5 %), with a household income between £19 542 and £30 107 (22·0 %), and with degree-level education or higher (47·2 %).

**Table 2.** Descriptive characteristics of a non-weighted and weighted sample in the SHeS21’s Intake24 sample

Characteristic	Number	Weighted percentage
Sex		
Female	1639	52·6
Male	1120	47·4
Age (years)		
16–24	122	10·4
25–34	315	16·4
35–44	408	15·9
45–54	485	17·5
55–64	630	17·4
65–74	593	13·4
75+	206	9
Ethnicity		
White	2665	94·5
Non-White	94	5·5
Income		
1 (>= £61 666)	617	21
2 (>= £42 774 < £61 666)	605	20·7
3 (>= £30 107 < £42 774)	526	17·4
4 (>= £19 542 < £30 107)	555	22
5 (>= £0 < £19 542)	456	18·9
Socio-economic classification		
Managerial and professional	1679	55·5
Intermediate	524	18·9
Routine and manual	556	25·6
Highest educational qualification		
Degree or higher	1515	47·2
HNC/D*	382	14·2
Higher grade*	386	16·3
Standard grade*	294	12·6
Other school level	54	2·9
No qualifications	128	6·7
SIMD quintile		
1 (most deprived)	317	17·5
2	477	20·7
3	571	19·1
4	722	21
5 (least deprived)	672	21·8
Urban–rural location		
Urban	2155	83·1
Rural	604	16·9
Region		
North	815	25·5
East	638	24·7
West	1306	49·9
Total	2759	100

SHeS21, Scottish Health Survey 2021; HNC/D, Higher National Certificate/Diploma; SIMD, Scottish Index of Multiple Deprivation.

* = or equivalent.

Table 2 also details the non-weighted numbers across groups for comparison.

### Consumption of ultra-processed food according to characteristics of participants

Overall, mean TEI among participants was 1637·8 kcal/d (95 % CI 1615·8, 1659·8). Mean UPF consumption was 919·9 kcal/d (95 % CI 901·1, 938·6), amounting to 55·4 % of TEI (95 % CI 54·7, 56·2). Mean UPF consumption as an absolute weight was 666·9 g/d (95 % CI 647·9, 685·9), equating to 28·2 % of total food weight.

UPF consumption varied across sociodemographic characteristics (Table 3). Males consumed more UPF than females (709·6 *v*. 628·4 g/d; 57·2 % *v*. 53·9 % TEI). UPF consumption was highest among younger age groups, peaking at 857·9 g/d (59·5 % TEI) in those aged 25–34 years and generally declined with increasing age. UPF consumption was higher among individuals from more deprived backgrounds, with those in the most deprived SIMD quintile consuming 62·1 % of TEI compared to 51·4 % in the least deprived. White participants and those living in urban areas also showed higher UPF consumption. Patterns across income and educational attainment were less consistent.

**Table 3.** Descriptive characteristics of UPF consumption (grams per day and %TEI) of a weighted sample in the SHeS21 Intake24 survey

Characteristic	UPF (g/d)	SD*	UPF (%TEI/d)	SD*
Sex				
Female	628·4	463·5	53·9	19·9
Male	709·6	530·3	57·2	20·1
Age (years)				
16–24	746·3	422·9	65·6	17·5
25–34	857·9	556·5	59·5	19·8
35–44	821·8	577·8	57·4	18·7
45–54	623·1	501·5	54·6	21·3
55–64	581·3	463·5	52·3	21·3
65–74	452·4	326·9	49·2	18·9
75+	433·6	284·5	50·1	16·1
Ethnicity				
White	675·1	500·6	55·9	19·9
Non-White	525	423·5	47·8	22·2
Income				
1 (>= £61 666)	654·9	487·5	52·6	19·9
2 (>= £42 774 < £61 666)	671·8	497·4	56·2	19·4
3 (>= £30 107 < £42 774)	700·2	493·3	55·6	19·9
4 (>= £19 542 < £30 107)	664·9	502·4	57·3	20·3
5 (>= £0 < £19 542)	646·4	508·6	55·5	20·7
Socio-economic classification				
Managerial and professional	618·1	436·6	53·5	19·8
Intermediate	675·8	533·6	55·1	19·9
Routine and manual	766	574·6	59·9	20
Highest educational qualification				
Degree or higher	625·5	468·2	51·8	19·5
HNC/D**	736·2	513·7	58·5	19·7
Higher grade**	785·4	550·8	58·7	20·3
Standard grade**	662·5	444·8	60·4	18·9
Other school level	536	508	56·2	22·3
No qualifications	587·1	553·6	56·9	21·2
SIMD quintile				
1 (most deprived)	755·1	506	62·1	20·6
2	774	607·6	58·4	20·9
3	651·9	481·9	55·1	19
4	585·6	421·8	51·6	18·9
5 (least deprived)	585·5	421·8	51·4	19
Urban–rural location				
Urban	685·8	509·7	56·1	20·3
Rural	574·2	423·4	52·1	18·4
Region				
North	575·8	493·4	51·8	20·4
East	576·5	457·4	50·8	20·7
West	642	516·8	54·3	19·4
Total	666·9	497·8	55·4	20·1

UPF, ultra-processed food; TEI, total energy intake; SHeS21, Scottish Health Survey 2021; HNC/D, Higher National Certificate/Diploma; SIMD, Scottish Index of Multiple Deprivation.

* sd.

** = or equivalent.

### Adjusted associations between ultra-processed food consumption and sociodemographic variables

#### Ultra-processed food consumption (g/d)

Results from the final models, incorporating all variables, indicate that sex, age, ethnicity, income, socio-economic classification, SIMD quintile and urban–rural location were associated with UPF consumption in absolute weight in grams per day (Table 4). Female (compared to male) (B = −80·3, *P* < 0·001), non-White (compared to White) (B = −291·1, *P* < 0·001) and rural (compared to urban) (B = −59·1, *P* = 0·020) participants had a lower UPF consumption (g/d).

**Table 4.** Adjusted linear regression analyses of UPF consumption (g/d and %TEI/d) by sociodemographic characteristics

Variables	UPF (%TEI)			UPF (g/d)		
	Unstandardised coefficients		Sig.	Unstandardised coefficients		Sig.
	B	Std. error		B	Std. error	
(Constant)	88·5	3·0	*P* < 0·001	1517·7	75·0	*P* < 0·001
Sex (ref. male)	−3·3	0·7	*P* < 0·001	−80·3	18·3	*P* < 0·001
Age	−2·8	0·2	*P* < 0·001	−73·2	5·4	*P* < 0·001
Ethnicity (ref. White)	−12·9	1·7	*P* < 0·001	−291·1	40·9	*P* < 0·001
Income	−0·2	0·3	*P* = 0·52	−15·3	7·5	*P* = 0·043
Socio-economic classification	0·9	0·5	*P* = 0·079	61·4	12·8	*P* < 0·001
Highest educational qualification	1·5	0·3	*P* < 0·001	−4·6	6·9	*P* = 0·51
SIMD quintiles	−1·8	0·3	*P* < 0·001	−36·2	7·5	*P* < 0·001
Rural location (ref. urban)	−1·5	1·0	*P* = 0·14	−59·1	25·4	*P* = 0·020
Region (ref. Western region)						
Northern region	−0·8	0·9	*P* = 0·41	19·4	23·4	*P* = 0·41
Eastern region	−1·8	0·9	*P* = 0·058	−30·1	22·9	*P* = 0·19

UPF, ultra-processed food; TEI, total energy intake; SIMD, Scottish Index of Multiple Deprivation.

UPF consumption (g/d) decreased with increasing age (B = −73·2, *P* < 0·001) and decreasing deprivation (i.e. higher SIMD quintiles) (B = −36·2, *P* < 0·001). Higher UPF consumption (g/d) was observed among individuals with higher income (B = −15·3, *P* = 0·043) and those in lower socio-economic classifications (B = 61·4, *P* < 0·001).

The regression model predicting UPF intake in grams per day was statistically significant (F = 36·7, *P* < 0·001), accounting for approximately 10 % of the variance (R^2^ = 0·1, adjusted R^2^ = 0·1) (Table 4).

#### Ultra-processed food consumption (percentage of total energy intake)

The model indicated that sex, age, ethnicity, highest educational qualification and SIMD quintile were associated with UPF consumption as a percentage of daily TEI (Table 4). A lower %TEI from UPF was consumed by participants who were female (compared to male) (B = −3·3 %, *P* < 0·001) and non-White (compared to White) (B = −12·9 %, *P* < 0·001). UPF consumption (%TEI) increased with decreasing education levels (B = 1·5, *P* < 0·001) and decreased with increasing SIMD quintiles, that is, decreasing deprivation (B = −1·8, *P* < 0·001).

Similarly, this model predicting UPF intake as a percentage of total energy (%TEI) was also significant (F = 38·2, *P* < 0·001), explaining approximately 10 % of the variance (R^2^ = 0·1, adjusted R^2^ = 0·1).

#### Distribution of energy intake (kcal) from ultra-processed food by food group

Food groups that contributed the most to participants’ TEI were ‘Cereal products, legumes and potatoes’ (357·0 kcal/d, 21·8 % of TEI), ‘Meat, fish and eggs’ (313·0 kcal/d, 19·8 % of TEI) and ‘Confectionery, cakes and biscuits’ (199·6 kcal/d, 11·6 % of TEI).

Similarly, most participants’ UPF calories (among participants reporting any UPF consumption, i.e. excluding individuals with no reported UPF consumption (weighted *n* 13)) came from the food groups ‘Cereal products, legumes and potatoes’ (244·8 kcal/d, 27·0 % of total UPF kcal), ‘Confectionery, cakes and biscuits’ (170·3 kcal/d, 17·9 % of total UPF kcal) and ‘Meat, fish, and eggs’ (153·6 kcal/d, 16·2 % of total UPF kcal).

**Figure 1.**
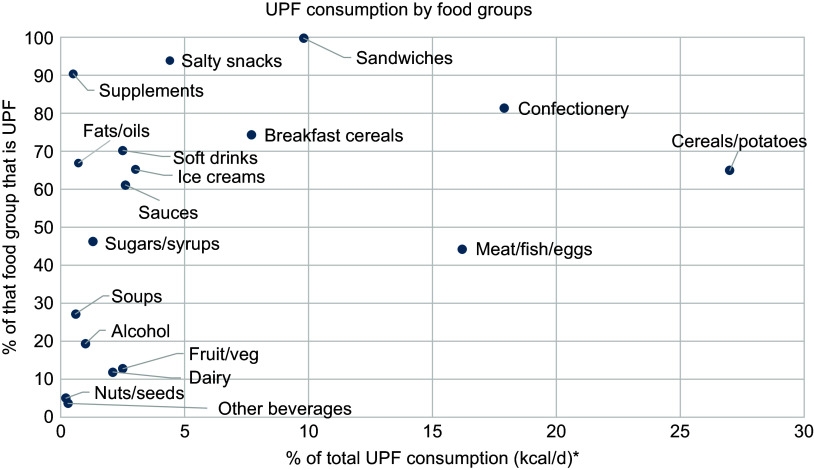
Description of energy intake (kcal) from UPF by food group per individual. * = from individuals who consumed UPF (weighted *n* 2632). Food groups with the highest UPF content are marked in bold. Food group names have been shortened in the figure for readability. UPF, ultra-processed food.

‘Sandwiches’ (99·8 %), ‘Salty snacks’ (94·1 %) and ‘Dietary supplements’ (90·5 %) were the food groups which contained the highest percentage of UPF (%TEI) amongst participants. All food groups contained at least some UPF, beginning from a low of 4·9 % of ‘Nuts and seeds’. Figure 1 outlines the distribution of UPF consumption by food group per individual.

## Discussion

### Key findings

From this cross-sectional study of a nationally representative sample of the Scottish population aged 16 years and over, average UPF consumption was 666·9 g/d, equating to 28·2 % of total food weight and 55·4 % of TEI.

Although mean TEI (1637·8 kcal/d) was lower than UK dietary reference values, similar levels have been reported in other UK studies using self-reported dietary recalls and are generally attributed to under-reporting^(45)^. Other studies analysing SHeS21 dietary data have also referenced this^(46)^.

### Interpretation of these findings in the current context

The level of UPF consumption parallels findings from similar studies which used dietary recall data from the NDNS to assess UPF intake across the UK’s adult population, in which mean UPF intake (%TEI) was found to be over half (*M* = 56·8 %^(2)^; *M* = 63 %^(22)^). This suggests that Scotland follows similar UPF consumption patterns to the rest of the UK.

As one systematic review demonstrates^(19)^, other HIC including Canada (*M* = 46–47 %), the USA (*M* = 57 %) and the Netherlands (*M* = 61 %), have similar levels of UPF consumption (%TEI) to Scotland. The high consumption of UPF in HIC is a symptom of their late phase in the ‘nutrition transition’^(1)^.

However, mean UPF consumption (%TEI) in Scotland appears to be higher than in some other highly urbanised HIC, including Italy (*M* = 17·3 %), Portugal (*M* = 23·8 %), South Korea (*M* = 24·9 %) and Chile (*M* = 28·6 %)^(19)^. Fewer studies have investigated the weight of UPF, but one study found that across the French population, UPF consumption as a percentage of total food weight was 18·4 %^(47)^. Social and cultural factors could explain this disparity. Countries like South Korea and Italy retain a strong home-cooking culture, with traditional processing methods like fermentation remaining widely practised in South Korea, exemplified by products like *kimchi* often being served at every meal^(48)^.

In contrast, Scotland has largely not retained a strong home-cooking culture^(49)^. Certainly, the fact that the consumption of unhealthy, deep-fried foods is widely considered part of Scottish national identity signifies this deviation from traditional cooking methods^(26)^. It is therefore perhaps unsurprising that UPF now make up over half of the national diet.

That UPF makes up only 28·2 % of total food weight, but 55·4 % of TEI, indicates its high energy density. These findings are useful to compare to Food Standard Scotland’s analysis of SHeS21, which found that ‘additional’ and ‘discretionary’ categories contributed to 28 % of TEI^(33)^. These government-defined categories only partially overlap with NOVA Group 4 foods and exclude some major contributors to UPF intake (e.g. packaged breads and processed meats). This suggests that existing policy measures targeting HFSS foods may substantially underestimate the true contribution of UPF to the Scottish diet. Addressing this gap by aligning policy categories more closely with UPF may strengthen future public health interventions.

The food groups with the highest percentage of UPF were ‘Sandwiches’, ‘Salty snacks’ and ‘Dietary supplements’. Dietary supplements including vitamins – which were not classified – protein powders and meal replacement shakes are predominantly UPF. Protein powders, in particular, have seen a surge in popularity, especially among younger, health-conscious consumers^(50)^.

Although these groups topped the list, all food categories contained some UPF, including ‘Fruits and vegetables’ and ‘Nuts and seeds’. This suggests that even foods typically considered minimally processed can be classified as UPF through additives, preservatives or flavour enhancers.

The food groups contributing the most UPF energy to diets were ‘Cereal products, legumes, and potatoes’, ‘Confectionery, cakes, and biscuits’ and ‘Meat, fish, and eggs’. Similar findings were observed in a Swiss study, likely due to the broad nature of these categories, which also account for the highest calorie intake^(21)^. This underscores that reducing UPF consumption requires more than cutting out snacks – it involves rethinking staple meal components. This challenges the Scottish Government’s classification of ‘additional’ or ‘discretionary’ categories, as UPF often forms a core part of daily diets.

Adjusted results revealed that a higher consumption of UPF (%TEI) was associated with male gender, White ethnicity, younger age, lower education, lower socio-economic classification and lower SIMD quintile. Again, these results parallel other studies assessing UK-wide UPF intake^(22,39)^. Regarding UPF weight (g/d), a higher consumption was associated with male gender, White ethnicity, younger age, higher income, lower socio-economic classification, higher deprivation and urban location. That more sociodemographic characteristics were associated with weight of UPF consumption (g/d) than UPF consumption (%TEI) could be since some UPF have a high weight, yet zero or few calories, such as artificially sweetened beverages.

Higher UPF consumption among more socio-economically disadvantaged groups may reflect greater affordability, availability and marketing of UPF, alongside structural constraints such as limited time, resources and facilities for home food preparation^(39)^. Differences by age, sex and ethnicity may further reflect variations in dietary norms, health awareness and exposure to food environments.

Importantly, this study’s findings contrast with FSS’s analysis of SHeS21, since they found a positive relationship between increasing age and increasing HFSS consumption^(33)^. Our findings have significant policy implications, as they suggest that policies to curb UPF consumption must be aimed at younger rather than older individuals.

#### Implications for policy and practice

The overall high consumption of UPF across Scotland invites policy changes.

### Education

First, the Scottish Government could consider adopting the NOVA classification system in their public health advice to emphasise the importance of consuming Group 1, 2 and 3 foods and avoiding Group 4 as much as possible. This has the potential to simplify their current rather complex and contradictory advice, making it easier for the public to follow. It could also simplify their current nutrient and food-specific national dietary targets. Like France, the Scottish Government could aim for a population-wide reduction of UPF consumption by 20 % during a 3-year period^(51)^.

Since both a young age and lower education were associated with high UPF consumption, the government should enhance culinary education throughout the school curriculum from primary school onwards in order to combat the ‘Cooking Skills Transition’^(49)^.

### Accessibility

Food procurement policies might also ensure that locally sourced MPF is used in school canteens and public sector workplaces. Since those living in areas of higher deprivation, on a lower income and of a lower socio-economic status consumed more UPF on average, the cost of MPF could be reduced by subsidisation, funded by UPF taxation.

Restricting the promotion of UPF is also key. The Scottish Government should extend their proposals to limit the promotional display of HFSS food and drinks in supermarkets to encompass all UPF, whilst, like Chile, the government could also prohibit the use of child-directed marketing, such as the use of cartoons and toys, for UPF products^(52)^. UPF products should not be disaggregated by food group for promotional restrictions, given that UPF was present across all food groups.

Overall, a holistic, multisectoral approach to curbing UPF consumption and improving public health is necessary. While adoption of NOVA is not without challenges – as it risks grouping a minority of nutritionally valuable products with nutrient-poor UPF and excludes certain unhealthy processed foods – it offers a more comprehensive framework than current HFSS-focused policy. With global precedent for its use in countries like France and Brazil, Scotland has an opportunity to build on this foundation and strengthen its public health strategy in line with international experience.

### Strengths and limitations

This study’s primary strength is that it is the first to quantify UPF consumption across Scotland in both g/d and %TEI, as well as offer an analysis according to sociodemographic measures and food groups. The use of SHeS21 was appropriate since it used individual-level dietary data, across two randomised days, and contained roughly equal numbers of individuals within each sociodemographic group. The sampling technique was rigorous, and it was able to produce a large sample size. Since the Scottish Government’s Intake24 weighting was used to account for non-response and sampling error, the results can be considered generalisable to the wider Scottish population.

The study’s reliability is enhanced by its use of freely accessible secondary data. Moreover, the process for classifying UPF consumption was informed by existing literature, meaning future researchers could follow the same classification. This also means that comparisons may be drawn to understand the differences in UPF consumption between Scotland and other countries, or how Scottish UPF consumption might change in the future.

However, the study is not without its limitations. First, finding that the average TEI was just 1637·8 kcal per d suggests that participants likely underestimated portion sizes, entered incorrect food descriptions or neglected to add certain food items.

Moreover, if UPF is selectively under-reported due to social desirability bias, this may lead to an underestimation of their contribution to TEI. However, misreporting is a common problem with recalls since they are subject to recall bias, and they remain widely accepted tools for population-level dietary assessment^(53)^. Despite lower absolute energy intakes, the proportion of energy derived from UPF closely mirrors estimates from other UK studies, supporting the internal consistency of the findings.

The coding of food groups, particularly for items like sandwiches, highlights challenges in accurately categorising UPF consumption, as participants often recorded composite dishes like sandwiches as single items, leading researchers to base coding decisions on the main ingredient (e.g. bread, typically UPF). This approach may overestimate UPF in some products and underestimate it in others, a common issue in food diary analysis, especially with datasets like NDNS that group multiple foods as composite dishes. To improve reliability, this study utilised established coding frameworks from previous studies, alongside resources like Open Food Facts and supermarket websites^(2,4,54)^.

### Future research

Given the association between a young age and high UPF intake, further studies should investigate this more by considering the Scottish adolescent population.

Whilst we calculated UPF consumption in grams per day, it might also be useful to consider UPF consumption as a percentage of total food intake in grams per day to test the hypothesis that UPF is more energy dense than non-UPF.

Although most agree that UPF is energy-dense and nutrient-poor, such that limiting it is likely beneficial, patterns of higher intake linked to obesity do vary by country^(55)^. Future research may examine which sociodemographic characteristics and other factors are linked to UPF consumption and its association with conditions such as obesity, within countries, so that public health advice can be tailored more specifically^(55)^.

## Conclusion

This study reveals that UPF dominates the Scottish diet, contributing over half of TEI and exhibiting stark sociodemographic disparities. The pervasive presence of UPF across all food groups highlights the limitations of current ‘discretionary food’ policies. Urgent action is needed to align Scotland’s public health strategies with international best practices, such as NOVA-based dietary guidelines, UPF taxation and marketing restrictions, particularly targeting younger and socio-economically disadvantaged populations. Addressing UPF consumption must be a cornerstone of efforts to reduce Scotland’s high burden of obesity and diet-related disease while promoting equitable access to MPF.

## Supporting information

10.1017/S1368980026102559.sm001 Curtis and Townsend supplementary material Curtis and Townsend supplementary material

## Supplementary material

For supplementary material/s referred to in this article, please visit https://doi.org/10.1017/S1368980026102559
