# Supplementary material for: Ultra-processed food consumption in Scotland: a nationally representative analysis of sociodemographic patterns and dietary contributions
Source: Public Health Nutr. 2026 Apr 30;29(1):e104. doi: 10.1017/S1368980026102559 (PMC13434613; doi:10.1017/S1368980026102559)
Supplement: Supplementary file 1 [file S1368980026102559sup001.docx]

**Supplementary Table 1: Food Group Conversion**

| **New Category (No.)** | **New Category Name** | **SHeS21 Codes** | **Original SHeS21 Categories** |
| --- | --- | --- | --- |
| 1 | Confectionery, cakes and biscuits | 43, 44, 7, 8, 9 | Sugar confectionery; Chocolate confectionery; Biscuits; Buns, cakes, pastries & fruit pies; Puddings |
| 2 | Meat, fish, and eggs | 22–35, 16 | Bacon & ham; Beef/veal & dishes; Lamb & dishes; Pork & dishes; Coated chicken; Chicken/turkey dishes; Liver & dishes; Burgers & kebabs; Sausages; Meat pies/pastries; Other meat/meat products; White fish coated/fried; Other white fish/shellfish/fish dishes; Oily fish; Eggs & egg dishes |
| 3 | Cereal products, legumes and potatoes | 38, 39, 59, 1–4 | Chips/fried/roast potatoes & products; Other potatoes/potato salads/dishes; Brown/granary/wheatgerm bread; Pasta/rice/other cereals; White bread; Wholemeal bread; Other bread |
| 4 | Juices and soft drinks | 61, 57, 58, 45 | Smoothies (100% fruit/juice); Soft drinks (not low calorie); Soft drinks (low calorie); Fruit juice |
| 5 | Dairy products | 10–14, 17, 60 | Whole milk; Semi-skimmed milk; Skimmed milk; Other milk & cream; Cheese; Butter; 1% fat milk |
| 6 | Added fats | 18–21 | PUFA margarine & oils; Low-fat spread; Other margarine/fats & oils; Reduced fat spread |
| 7 | Salty snacks | 42 | Crisps & savoury snacks |
| 8 | Sugar, honey, jam, sweet sauces and syrups | 41, 55 | Sugars/preserves/sweet spreads; Artificial sweeteners |
| 9 | Breakfast cereals | 5, 6 | High fibre breakfast cereals; Other breakfast cereals |
| 10 | Ice-creams and milk-based desserts | 53, 15 | Ice cream; Yogurt, fromage frais & dairy desserts |
| 11 | Alcoholic beverages and substitutes | 47, 48, 49 | Spirits & liqueurs; Wine; Beer/lager/cider/perry |
| 12 | Soups and broth | 50C, 50D | Soup (manufactured); Soup (homemade) |
| 13 | Nuts and seeds | 56 | Nuts & seeds |
| 14 | Fruits and vegetables | 36, 37, 40 | Salad & other raw vegetables; Vegetables not raw; Fruit |
| 15 | Savoury sauces, pickles, gravies & condiments | 50R | Savoury sauces, pickles, gravies & condiments |
| 16 | Other beverages including tea, coffee, hot chocolate | 50A, 51 | Beverages (dry weight); Tea, coffee & water |
| 17 | Dietary supplements | 54, 50E | Dietary supplements; Nutrition powders & drinks |
| 18 | Sandwiches | 62 | Sandwiches |

Items in SHeS category 52 (“Commercial Toddlers’ Foods and Drinks”), were individually coded into new food groups.
